# Prioritizing Melanoma Surgeries to Prevent Wait Time Delays and Upstaging of Melanoma during the COVID-19 Pandemic

**DOI:** 10.3390/curroncol30090604

**Published:** 2023-09-09

**Authors:** Katherine Aw, Rebecca Lau, Carolyn Nessim

**Affiliations:** 1 Faculty of Medicine, University of Ottawa, 541 Smyth Road, Ottawa, ON K1H 8M5, Canada; 2 The Ottawa Hospital Research Institute, The Ottawa Hospital, 501 Smyth Road, Ottawa, ON K1H 8L6, Canada; 3 Department of General Surgery, Division of Surgical Oncology, The Ottawa Hospital, 501 Smyth Road, Ottawa, ON K1H 8L6, Canada

**Keywords:** melanoma, COVID-19, melanoma surgery, wide local excision, wait time, cancer staging

## Abstract

Prompt diagnosis and surgical management of melanoma strongly impact prognosis. Considering the limited resources, emergency closures, and staffing shortages during the COVID-19 pandemic in Canada, our institution implemented a dedicated care pathway to prioritize cancer surgeries. We aim to assess whether this strategy was effective at preventing surgical wait time delays and upstaging of melanoma. We retrospectively collected data of patients aged ≥18 years with biopsy-proven primary melanoma who underwent wide local excision (WLE) ± sentinel lymph node biopsy (SLNB) between 1 March 2018–29 February 2020 (pre-pandemic) and 1 March 2020–22 March 2022 (pandemic). Patients with distant metastasis, recurrence, in situ disease, and unknown primary were excluded. Wait time from consult to surgery, tumour (T) and nodal (N) stage, and overall stage were collected. Results: We included 419 patients [pre-pandemic (*n* = 204) and pandemic (*n* = 215)]. Median wait time (days) [interquartile range] to surgery was 36 [22–48] pre-pandemic and 35 [24–49] during the pandemic (*p* = 0.888). There were no differences found in T stage (*p* = 0.060), N stage (*p* = 0.214), or overall melanoma stage (*p* = 0.192). We highlight the importance of streamlining melanoma surgery during a pandemic. As the need arises to meet surgical backlogs including benign surgery, dedicated cancer surgery should maintain a priority to not negatively affect cancer outcomes.

## 1. Introduction

The COVID-19 pandemic has resulted in major disruptions to the delivery and utilization of healthcare services. Globally, healthcare systems saw reductions in patient visits, diagnostic testing and imaging, and therapeutic care during the pandemic [1]. A 2020 global expert response study projected that over 28 million elective surgeries would be cancelled over the 12 weeks of peak disruption due to COVID-19, with a projected 37.7 percent of cancer surgeries postponed or cancelled [2]. For patients with cancer, surgery continues to be a mainstay of treatment. However, the pandemic and the associated implementation of public health restrictions have presented barriers to timely access to surgical care. In fact, in a recent international prospective study involving 15 tumour types and 61 countries, it was estimated that one in seven patients were unable to undergo scheduled surgeries due to COVID-19 pandemic lockdowns [3].

The Canadian Cancer Society estimates that 8700 Canadians are diagnosed with melanoma skin cancer and 1250 Canadians will die of melanoma each year [4]. The most commonly performed surgical procedures for primary cutaneous melanoma are wide local excision (WLE), sentinel lymph node biopsy (SLNB), and lymph node dissection (LND), which allow for subsequent staging of the disease.

For aggressive cancers such as melanoma, early diagnosis, staging, and treatment play a significant role in prognosis and patient survival. More advanced T and N stages in melanoma are associated with worse disease-free survival and a higher risk of recurrence [5,6]. While surgical excision in patients with melanoma that is less than 1 mm in depth and localized to the skin have a 93–97% 5-year survival rate, patients with late-stage distant metastatic melanoma only have a 10–20% 5-year survival rate, depending on the location of the metastasis [5].

To help guide treatment decisions, improve patient survival, and maintain quality of care, Cancer Care Ontario (CCO) recommends utilizing the Wait Time Information System (WTIS) which assigns priority levels and target times to patients with cancer [7]. Patients categorized under Priority 1 require emergency surgery with a target time from the decision to treat to having cancer surgery of within 24 h [7]. Patients categorized under Priority 2 are diagnosed with highly aggressive malignancies and should undergo surgery within 14 days [7]. Patients labelled Priority 3 have known or suspected invasive cancer not meeting criteria for Priority 2 or Priority 4 and have a target time to surgery of 28 days [7]. Lastly, patients labelled Priority 4 are diagnosed with indolent malignancies and are recommended to receive surgery within 84 days [7].

In Ontario, Canada, studies have reported major reductions in melanoma diagnoses with incidence rates decreasing by more than 50% (2016–2020) and a 27% decline in the volume of skin biopsies performed during the pandemic (2019–2020) [3,4]. A study from Alberta, Canada found a 43% reduction in melanoma diagnoses during the COVID-19 pandemic and estimated that an extra 223 melanomas would have been diagnosed at their centre between March and December 2020 had the pandemic not occurred [8]. Fu et al., 2023, have also reported worse short-term survival for Ontario melanoma patients during the pandemic in 2020 [9]. Several international studies have also shown that patients have presented with higher-stage melanoma since the start of the pandemic [10,11,12,13,14]; however, there is a scarcity of studies assessing wait times to melanoma surgery during the COVID-19 pandemic. No studies to date have explored the impact of the COVID-19 pandemic on melanoma surgical management, wait times, and staging in Canada.

The COVID pandemic has fueled great discussion about the need to safeguard surgical pathways in efforts to cope with resource strains created by public healthcare crises [1,2]. The COVIDSurg collaborative, of which our institution is a member, has advocated for the prioritization of cancer surgeries to prevent delays during a pandemic [1,2]. In fact, a four-week delay to receiving cancer surgery was associated with an increased mortality risk [10].

In light of the pandemic, the CCO created updated guidelines during the pandemic on wait time management for all cancer disease sites and treatment programs, including genetics clinics, palliative care symptom management, radiation treatment, surgical oncology, and systemic therapy [11]. For melanoma, CCO defined surgical patient populations as priority A, B, or C [11]. Under this system, patients in WTIS Priority 1 and 2, and some Priority 3 patients with emergent and very aggressive tumours, are re-classified as Priority A [11]. A delay in surgery for these Priority A patients would result in immediate threat to life or would significantly change the patient’s prognosis [11]. Patients classified as WTIS Priority 3, and some Priority 4 tumours, are labelled Priority B [11]. A delay of less than four weeks from target for Priority 3 patients would not be anticipated to significantly impact survival or outcome [11]. Priority C includes WTIS Priority 4 patients with indolent tumours for whom a delay of eight weeks would be unlikely to impact outcome [11].

During the COVID-19 pandemic, our institution cancelled all elective surgeries for benign surgeries and adopted this CCO surgical patient prioritization system in an attempt to maintain timely surgical management for melanoma patients. We aim to assess whether these dedicated care pathways that prioritized melanoma surgeries prevented wait time delays and upstaging in melanoma. The purpose of our study is to compare wait time and tumour (T) stage, nodal (N) stage, and overall staging of melanoma in patients before the pandemic (1 March 2018–29 February 2020) and during the pandemic (1 March 2020–22 March 2022).

## 2. Materials and Methods

### 2.1. Study Design

**Figure 1:**
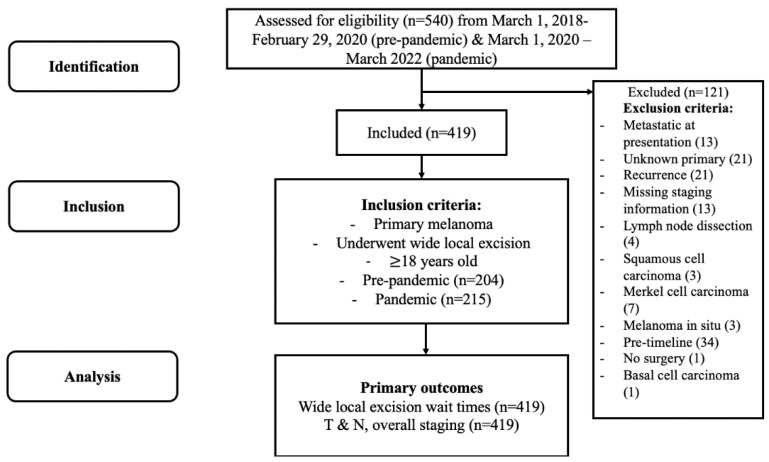
Schematic highlighting study workflow.

Patients who received a consultation for melanoma surgery between 1 January 2018 and 22 March 2022 were identified retrospectively in a melanoma database at the Ottawa Hospital, a tertiary care center affiliated with the University of Ottawa (Figure 1). Patients were mainly referred for surgical consultation by dermatologists, medical oncologists, or primary care physicians. Patients were included if they were ≥18 years old, had biopsy-proven primary melanoma, and underwent WLE during the study timeline of 1 March 2018–29 February 2020 (pre-pandemic) and 1 March 2020–22 March 2022 (pandemic). This timeline was selected to make both cohorts comparable in time period, where March 2020 corresponded to the announcement of pandemic shutdowns in Ontario. Patients with distant metastasis, recurrence, unknown primary, and missing staging information were excluded. The primary outcomes collected were wait time, as defined by time from surgical consult to definitive WLE surgery, T stage, N stage, and overall melanoma stage. Patient demographics, including age, sex, Charlson Comorbidity Score (CCS), and tumour histological characteristics were also collected.

Of the 540 patients identified, 121 patients were excluded (Figure 1). Thirty-four patients were excluded as they presented before the defined timeline of inclusion. Patients with unknown primary melanoma (*n* = 21), recurrence (*n* = 21), metastatic disease (*n* = 13), non-melanoma skin cancer (*n* = 11), melanoma in situ (*n* = 3), or metastatic disease at presentation (*n* = 13) were also excluded. One patient did not undergo planned surgery, and four patients had already undergone WLE for the primary tumour before the study’s timeline and presented during the study timeline for LND only. 

### 2.2. Statistical Analysis

SPSS (Version 28, IBM, Armonk, NY, USA) statistical software was used to conduct univariable descriptive statistics (frequencies, proportions, medians, and interquartile ranges). Continuous variables were analyzed with the Mann–Whitney U Test (Wilcoxon rank sum). Fischer’s exact test was performed to compare proportional differences between categorical variables. Categorical variables with contingency tables greater than 2 × 2 were analyzed using the Pearson chi-squared test. A subsequent two-sample z-test was used to identify differences in column proportions for each subset of the categorical variable, and *p*-values were adjusted using the Bonferroni method. Missing data were addressed using a pairwise deletion approach. The threshold for statistical significance was set to *p* < 0.05.

## 3. Results

### 3.1. Study Population

A total of 419 patients were included for analysis (Table 1). There were 204 patients in the pre-pandemic cohort and 215 patients in the pandemic cohort. The median age (years) [interquartile range] was 68.0 [60.0–76.5] pre-pandemic and 64.0 [55.0–74.0] in the pandemic group (*p* = 0.017). The M:F ratios for the pre-pandemic and pandemic groups were 1.3:1 and 1.2:1, respectively. The total CCS was significantly different between groups (*p* = 0.007). Although the median CCS was similar (3 [3-4] pre-pandemic and 3 [3-3] during the pandemic), patients in the pandemic cohort seemed to present with fewer comorbidities; there were more patients in the pandemic cohort with CCS scores of 0–2 [pre-pandemic (*n* = 28) and pandemic (*n* = 43)]. Compared with the pre-pandemic group, more SLNBs were performed (*p* = 0.039) in the pandemic cohort. The frequency of LNDs completed was comparable between the pre-pandemic and pandemic groups.

The histological type of melanoma did not differ between groups, with superficial spreading, nodular, and not otherwise specified as the most common melanoma types in both groups (Table 2). Overall, there was no difference in the location of melanoma. When looking at specific locations, however, the pre-pandemic group had more head and neck tumours (*p* < 0.05), which may also explain the lower rate of lentigo maligna melanoma during the pandemic (*p* < 0.05). Breslow thickness, presence of ulceration, and mitotic index did not differ between groups.

### 3.2. Wait Times

There were no differences in overall wait time from consult to WLE surgery date between cohorts, with the pre-pandemic group and pandemic group having wait times (days) [IQR] of 35.5 [22.0–48.0] and 34.5 [24.0–49.0], respectively (*p* = 0.888) (Table 3). When comparing wait times by overall TNM melanoma stage, only stage-IIA disease had a longer wait time of 34.0 [25.0–48.0] days in the pandemic group when compared with the pre-pandemic group, who experienced a wait time of 25.0 [13.0–40.0] days.

### 3.3. Tumour, Nodal, and Overall Staging

The frequency of T stage, N stage, and overall stage did not differ between groups (Table 4). Within T, N, and staging sub-analyses, a greater proportion of T2b melanomas was detected in the pandemic group (4.7%) when compared with the pre-pandemic group (1.0%) (*p* < 0.05). More stage-IIID disease was identified in the pre-pandemic group (6.4%) compared with the pandemic group (1.9%) (*p* < 0.05).

## 4. Discussion

### 4.1. Key Findings

To our knowledge, there are no existing studies that assess the impact of the COVID-19 pandemic on melanoma surgical wait times and staging in Canada. In this study, we found no increase in wait times or upstaging of melanoma in patients undergoing WLE + SLNB during the pandemic when compared with pre-pandemic. The wait times between the pre-pandemic and pandemic cohorts (35.5 days [22.0–48.0] and 34.5 days [24.0–49.0], respectively) were not significantly different. There were no differences in overall T and N staging between groups. Our findings highlight how implementing a prioritized care pathway for cancer surgeries can prevent hospitals from compromising melanoma surgical care during a pandemic.

Existing literature suggests that melanoma overall staging and histological prognostic features have worsened since the beginning of the COVID-19 pandemic [12,13,14,15,16,17,18,19,20]. For instance, a four-year Romanian study (2018–2022) that did not report the use of a prioritization system showed higher Breslow thickness and more stage-III patients during the pandemic [20]. In contrast, in the current study, where we implemented melanoma surgery prioritization, we report no difference in histological features and no upstaging of melanoma. Some other existing studies, including one by Demacrel et al., have similarly reported no increases in tumour thickness over the pandemic [21,22,23]. Use of melanoma surgery prioritization, however, are unclear or varied amongst these studies.

The current study did not find any differences in surgical wait times before and during the pandemic. Given the aggressive nature of melanoma, it is important for patients, especially those with more advanced disease, to receive prompt surgical treatment. We show that there were no differences in wait time by stage between the pre-pandemic and pandemic cohorts, with the exception of patients with stage-IIA disease, who experienced a 9-day delay during the pandemic. This difference in wait time for stage-IIA patients was statistically but not clinically significant and was within the acceptable delay of 4 weeks defined by the CCO pandemic guidelines. Encouragingly, our patients with the most advanced melanomas, stage-IIIA–IIID patients, did not experience any delays, which further supports the need to prioritize melanoma surgeries during a pandemic.

Existing studies that also implemented surgical prioritization for melanoma have similarly reported no difference in wait time or even a reduction in wait times during the pandemic [19,24]. In a study involving 12 Italian institutions that prioritized melanoma surgeries over other skin surgeries, patients maintained a two-week maximum surgical wait time for melanoma during the pandemic, which was the same wait time as pre-pandemic [19].

In comparison, an England study found an initial reduction in wait times for melanoma treatment by 58% in May 2020 followed by an increase in wait times by up to 91% by December 2020 [25]. This study did not report any melanoma surgery prioritization, which may explain the increase in wait times during the pandemic, particularly in December 2020, which coincided with the implementation of lockdown restrictions. A reduction in melanoma diagnoses during the pandemic may have contributed to the initial decrease in pandemic wait times in this study. In fact, numerous studies have shown a reduction in melanoma diagnoses during the pandemic, ranging from 18–86% [15,16,17,26,27,28,29,30,31].

Upstream of surgical management, diagnostic and referral delays may serve as additional contributors to melanoma patients presenting later in their disease course. Aebed and colleagues reported a decrease in the number of patients seeking care for melanoma during the two lockdown periods in Romania and a delay in asking for a medical opinion by six to nine weeks during the pandemic [20]. Furthermore, Johnstone and colleagues found that mean wait time for routine and urgent referrals to dermatology for melanoma increased in 2021 during the pandemic [32]. As such, patient factors and timeliness of primary and secondary care services may also influence a patient’s clinical course of disease and management.

### 4.2. Study Limitations

The current study has several limitations. First, this study is limited by its retrospective design, which is susceptible to selection bias. Given that the study was conducted in a single hospital, it is also challenging to generalize our findings to other countries and healthcare institutions where public health measures during the pandemic may have differed significantly. Additionally, we did not assess long-term outcomes including overall survival and disease-free survival. We did not analyze our data for temporal changes, which limited our ability to capture monthly or year-to-year fluctuations in our variables of interest that may have occurred during lockdown periods and subsequent waves of the pandemic. Furthermore, the timeline may not be sufficiently long enough to capture any upstaging of disease. Although we collected data on patients with stage-I–III melanoma, we did not collect data on patients initially presenting with stage-IV metastatic disease, as they were not surgical candidates.

### 4.3. Implications and Future Directions

Our study supports the idea that the implementation of a dedicated care pathway for cancer surgeries during the COVID-19 pandemic prevented surgical wait time delays and upstaging of melanoma. Early detection and treatment of melanoma has been well established as a predictor of favourable patient prognosis and overall survival [5,6].

Beyond improving prognosis and survival in melanoma patients, access to prompt surgical management of melanoma during a pandemic should remain a priority to not negatively impact the mental state of patients with melanoma. A multi-centre prospective study in France used validated questionnaires to assess the psychological impact of treatment modifications during and after the COVID-19 lockdown on patients with breast cancer and gynecological cancers [33]. This study found that quality of life and psychological state were impaired during COVID-19 lockdowns, with a significantly higher number of confirmed anxiety cases in patients for whom treatment was delayed or cancelled [33]. In patients with melanoma specifically, a recent cross-sectional study assessed psychological outcomes during the COVID-19 pandemic in China and found that patients with melanoma experienced heightened fear of progression, increased depression, and elevated anxiety during the pandemic [34].

In addition to patient care, it is important to consider the economic consequences of treatment delays, particularly on publicly funded healthcare systems. For example, a study looking at stage-specific treatment costs of melanoma in Ireland reported that the cost of treatment for stage-IV melanomas was 25-fold higher than the cost of treating stage-IA disease [35]. Furthermore, two systematic reviews also emphasize the massive financial burden of melanoma and highlight the fact that advanced-stage disease, especially stages III–IV, are associated with higher costs compared with the general population [36,37]. With surgery serving as a mainstay intervention for early-stage disease, it remains crucial for patients to undergo planned surgeries in a timely fashion to prevent disease progression and to mitigate costs to the healthcare system.

As we emerge from the pandemic and prioritization measures lift, we anticipate surgical backlogs from the reinstation of elective surgeries for benign disease and, thus, longer wait times for melanoma surgery. There have been predictive models of surgical backlogs to be anticipated post-pandemic. Further data collection is being undertaken currently by this study group with a longer timeline to assess for long-term outcomes, including survival and mortality rates as well as any upstaging of disease during this post-pandemic era, as this backlog persists and dedicated pathways are no longer in place. Looking ahead to the future, our findings could help inform surgical care policies and measures during public health care crises. Ultimately, cancer surgeries should remain a priority to maintain quality of care and prevent the worsening of cancer outcomes.

## Figures and Tables

**Table 1:** Patient demographics.

Characteristic	Pre-Pandemic (*n* = 204)	Pandemic (*n* = 215)	*p*-Value
**Age, years, median (IQR)**	68.0 (60.0–76.5)	64.0 (55.0–74.0)	0.017
**Sex, *n* (%)**			0.624
Male	117 (57.4)	118 (54.9)	
Female	87 (42.6)	97 (45.1)	
**CCS, median (IQR)**	3 (3-4)	3 (3-3)	0.007
**CCS, *n* (%)**			
0–2	28 (13.7)	43 (20.0)	
3–5	166 (81.4)	160 (74.4)	
6–8	10 (4.9)	12 (5.6)	
**Palpable disease at presentation, *n* (%)**	14 (6.9)	11 (5.1)	0.538
**SLNB performed, *n* (%)**	183 (89.7)	205 (95.3)	0.039
**LND performed, *n* (%)**	26 (12.7)	17 (7.9)	0.110

IQR, interquartile range; CCS, Charlson Comorbidity Score; SLNB, sentinel lymph node biopsy; LND, lymph node dissection.

**Table 2:** Tumour characteristics and histological features.

Characteristic	Pre-Pandemic (*n* = 204)	Pandemic (*n* = 215)	*p*-Value
**Histological type, *n* (%)**			0.094
Superficial spreading	78 (38.2)	97 (45.1)	
Nodular	57 (27.9)	59 (27.4)	
Not otherwise specified	35 (16.6)	31 (14.4)	
Lentigo maligna	12 (5.9) *	3 (1.4) *	
Desmoplastic	5 (2.5)	7 (3.3)	
Acral lentiginous	3 (1.5)	3 (1.4)	
Spitzoid	0	2 (0.9)	
Mixed	5 (2.5)	3 (1.4)	
Not reported	5 (2.5)	1 (0.5)	
Other	4 (2.0)	9 (4.2)	
**Location, *n* (%)**			0.271
Head and neck	40 (19.6) *	25 (11.6) *	
Back	43 (21.1)	52 (24.2)	
Trunk	21 (10.3)	20 (9.3)	
Arm	40 (19.6)	43 (20.0)	
Leg	29 (14.2)	39 (18.1)	
Shoulder	12 (5.9)	15 (7.0)	
Finger	0	2 (0.9)	
Scalp	10 (4.9)	9 (4.2)	
Foot	4 (2.0)	8 (3.7)	
Toes	3 (1.5)	1 (0.5)	
Vulva/Vagina	0	1 (0.5)	
Other	2 (1.0)	0	
**Breslow thickness, mm, median (IQR)**	1.7 (1.0–3.1) *n* = 202	1.7 (1.0–3.0) *n* = 213	0.968
**Ulceration, *n* (%)**	55 (27.8) *n* = 198	52 (24.5) *n* = 212	0.459
**Mitotic index, mitoses/mm^2^, median (IQR)**	2.0 (1.0–5.0) *n* = 197	2.0 (1.0–5.0) *n* = 209	0.453

IQR, interquartile range; mm, millimeter. * Denotes a difference (*p* < 0.05) in column proportions for this subset of the categorical variable.

**Table 3:** Overall wait times and wait times by melanoma stage.

Characteristic	Pre-Pandemic (*n* = 204) ^1^	Pandemic (*n* = 215) ^2^	*p*-Value
**Wait time ^3^, days, median (IQR)**	35.5 (22.0–48.0)	35.0 (24.0–49.0)	0.888
**Wait time by stage, days, median (IQR)**			
IA	38.5 (23.0–49.5)	43.0 (30.0–51.5)	0.270
IB	29.0 (19.0–43.0)	32.0 (20.0–43.0)	0.818
IIA	25.0 (13.0–40.0)	34.0 (25.0–48.0)	0.048
IIB	40.0 (24.0–44.0)	41.0 (31.0–59.0)	0.169
IIC	43.0 (30.0–57.0)	32.0 (23.0–46.0)	0.201
IIIA	29.0 (18.0–35.0)	35.0 (30.0–49.0)	0.297
IIIB	43.0 (28.0–47.0)	36.0 (26.0–61.0)	0.926
IIIC	39.0 (29.0–60.0)	30.0 (21.5–40.0)	0.139
IIID	31.0 (23.0–48.0)	29.0 (14.0–43.0)	0.624

IQR, interquartile range. ^1^ Sample sizes for each stage in the pre-pandemic group are as follows: IA (*n* = 48); IB (*n* = 41); IIA (*n* = 19); IIB (*n* = 21); IIC (*n* = 15); IIIA (*n* = 9); IIIB (*n* = 9); IIIC (*n* = 29); IIID (*n* = 13). ^2^ Sample sizes for each stage in the pandemic group are as follows: IA (*n* = 48); IB (*n* = 57); IIA (*n* = 25); IIB (*n* = 13); IIC (*n* = 13); IIIA (*n* = 9); IIIB (*n* = 14); IIIC (*n* = 32); IIID (*n* = 4). ^3^ Wait time is defined as days from consult to definitive wide local excision surgery date.

**Table 4:** Tumour, nodal, and overall staging information.

Characteristic	Pre-Pandemic (*n* = 204)	Pandemic (*n* = 215)	*p*-Value
**T stage ^1^, *n* (%)**			0.060
T1a	10 (4.9)	18 (8.4)	
T1b	42 (20.6)	33 (15.3)	
T2a	47 (23.0)	64 (29.8)	
T2b	2 (1.0) *	10 (4.7) *	
T3a	27 (13.2)	30 (14.0)	
T3b	19 (9.3)	12 (5.6)	
T4a	17 (8.3)	15 (7.0)	
T4b	40 (19.6)	33 (15.3)	
**N stage ^1^, *n* (%)**			0.214
N0	144 (70.6)	159 (74.0)	
N1a	25 (12.3)	21 (9.8)	
N1b	2 (1.0)	3 (1.4)	
N1c	3 (1.5)	8 (3.7)	
N2a	10 (4.9)	9 (4.2)	
N2b	2 (1.0)	0	
N2c	2 (1.0)	6 (2.8)	
N3a	1 (0.5)	2 (0.9)	
N3b	3 (1.5)	0	
N3c	12 (5.9)	7 (3.3)	
**Stage ^1^, *n* (%)**			0.192
IA	48 (23.5)	48 (22.3)	
IB	41 (20.1)	57 (26.5)	
IIA	19 (9.3)	25 (11.6)	
IIB	21 (10.3)	13 (6.0)	
IIC	15 (7.4)	13 (6.0)	
IIIA	9 (4.4)	9 (4.2)	
IIIB	9 (4.4)	14 (6.5)	
IIIC	29 (14.2)	32 (14.9)	
IIID	13 (6.4) *	4 (1.9) *	

All patients included had M0 disease. ^1^ All melanomas were staged according to the eighth edition of the American Joint Committee on Cancer staging system. * Denotes a difference (*p* < 0.05) in column proportions for this subset of the categorical variable.

## Data Availability

The data presented in this study are available upon request from the corresponding author. The data are not publicly available due to privacy and ethical constraints.
